# The Treatment of the Insane

**Published:** 1907-03-30

**Authors:** G. H. Savage

**Affiliations:** Physician for and Lecturer on Mental Diseases, Guy's Hospital


### Hospital Clinics.

### THE TREATMENT OF THE INSANE.

By DR. G. H. SAVAGE, M.D.(Lond.), F.R.C.P. (Lond.), Physician for and Lecturer on Mental Diseases, Guy's Hospital.

(A Lecture delivered at the Polyclinic.)

### Historical Review.

I am not quite sure whether the name given to this address is right. First of all I think it should, perhaps, be on the more recent treatment of the insane. Again, I am always protesting against treating insanity as a definite disease. We have to consider that the treatment of the insane differs very materially from what it was even a very few years ago. A knowledge of the history of their treatment in the past is always essential if we would understand where we have reached now. The rate of recovery is not now so much greater than what it was 150 years ago, which, of course, one regrets, and hopes for better things. The treatment even in the long past—I say frequently—even the Biblical treatment of Saul by David was certainly correct, and the treatment of Nebuchadnezzar by turning him into the country until he recovered was essentially good. We have only come back to this even now. The next idea was that all insanity was associated with theological error, that, in fact, every insane person was affected by some spirit—a spirit generally of evil, but sometimes a prophetic spirit. There the only way was to eject the evil spirit by casting it out, securing with chains, chastening with whips; other methods also were used for this purpose. Then there is the interesting survival where the evil spirit had to be cast out by noxious smells and other remedies, and in these cases remedies like asafœtida were used for the ejection of the devil. All of which are extremely interesting; there is still existing in the centre of Europe, in Gheel, the city of the Simple, a cathedral where for 700 years the patients have been treated at the shrine of Dymphna on the theological principles. I may say that here I spent a week of great interest years ago. Again, I am now asked, on an average once a week, whether there is any objection to such and such a patient being treated for insanity by Christian science. The orthodox and justifiable treatment by hypnotism I am also daily being asked about, and I shall refer to it later.

### The Development of Asylums.

Subsequent to this early treatment, society, observing more closely, said, "We must be protected against the accidents that result from the insanity of certain people. In fact, society must protect itself. Shut up the insane." So asylums grew, and are growing still. And, of course, in the earlier days asylums were not specialised. Patients were all classed as people of unsound mind, whether they were idiotic, imbecile, or maniacal; curable and incurable were classed together. To-day one of the chief developments in the treatment of the insane is the separation of these classes, so that now not only have you idiot asylums, but even these are being separated, so that you have schools for the feeble-minded, and institutions and colonies for those who are weak-minded, but not distinctly idiotic.

Next, and I think this is one of the developments that will have a very great influence in the future, came the establishment of hospitals for the insane. I have said to many of you probably before that there are two words I should like to get rid of in the English language—"asylum" and "lunatic." It will take a hundred years to do away with the stigmata implied in these words—the feeling that because a person is affected in his mind therefore he is alien and must be shut off, so that a man suffering from disease of his highest faculties is treated as an outcast. When one comes to remember that a large proportion of these people get well, and that many of them remain well for the rest of their lives, it is a shame that they should be treated as altogether useless. Let me remind you that at the present moment in Glasgow, and we must admit that North of the Tweed they are in advance of us in many ways, that in Glasgow they have now a receiving hospital so that every patient suffering from mental disorder who falls into the hands of the equivalent of the relieving officer or the police, is sent to this hospital and not to the infirmary or asylum right away, unless he happens to be a typical case of general paralysis or some incurable disease of that kind, in which case he may at once be transferred to the asylum. Otherwise there are two wards into which the cases are sent, and I have spent a day there not long ago to see the types of cases and to see the results. It appears that more than half of these patients never go into asylums at all, and are discharged. I suppose some of you would surmise that drink is the cause of many of these admissions. It is a cause for a considerable number, but does not by any means cover the majority of the cases. And even in this case the superintendent told me, when I was sceptical about the recovery of the acute alcoholics, that they did not come back frequently.

The experience proves that treatment in a hospital like this for a month or six weeks is quite as good and as useful in the long run as detention for two or three months in an asylum. In fact, unless the inebriate is going to be secluded for a year or two, the hospital treatment in bed and supervision of diet and medicine, to a certain extent, are quite as efficacious as a prolonged treatment, and much more useful than a simple detention for three or four months. You will understand, therefore, why I have spoken of the development of treatment along this line.

### Voluntary Boarders.

As I have pointed out, another line that is very instructive in its development is the hospital-asylum treatment, a type of which Bethlem Hospital is the oldest. St. Luke's is another old institution, and scattered all over the country in our day you have sanatoria as, for example, Hayling Sanatorium. What a salvation there is in that word instead of "asylum." People do not mind going to a sanatorium. In York they have the "retreat." People do not mind going to a "retreat." There are hospitals at Exeter, Gloucester, Manchester, indeed, scattered all over the place you have hospitals for the insane, and they are, of course, self-supporting to a great extent. In these certain patients pay well and others pay very little, so that those who pay more than the cost help to support those who pay less. At Bethlem Hospital the majority pay nothing at all, whereas certain other patients pay two guineas a week. Other asylums receive patients for one guinea a week. All these are important developments. There is another mode in which I feel particularly interested—I myself, in fact, revived the voluntary boarder system. At that time there was an opportunity for the royal hospitals like Bethlem to receive a certain number of patients suffering from mental disorder as voluntary boarders. I had difficulties and contentions with the authorities on this point. They said, "This man is of unsound mind; he has got delusions; can you receive as voluntary boarders patients suffering in that way?" Replying in the affirmative, I was asked if the patient could not be certified. My answer was, "Yes, but we do not want him to be certified." If a patient came to me and said, "I am supposed to be of unsound mind. I do not think I am; anyway, I am quite willing to go into a hospital where I can be under observation and where you can see who is wrong and who right, and you can determine whether I am a person suffering from mental disorder." The patient showed a preference for coming to these places, and in the old days we frequently had patients arriving at Bethlem Hospital saying, "I feel wrong in my head and I want to be taken in." Now at Bethlem there are about 25 voluntary boarders, and at Virginia Water also a large number of similar patients, and one hopes that this system will develop still further. You can understand, of course, that in the county asylums and other similar places it would be rather a dangerous thing to have voluntary boarders, but at present, patients who recognise the importance of being under care and treatment, may go to hospitals like those at Virginia Water and Bethlem, and ask for admission. But the Commissioners required the authorities, quite properly, to have in writing the voluntary statement, and at the same time there must be a statement by an outside medical man preferably a general practitioner, that in his judgment the patient is a fit and proper one. Over and over again such a case as this occurs. A man presents himself saying the whole world is against him and he will kill himself. I say, "You feel you could kill yourself; very well, don't. It will be very inconvenient for your friends and for me. I will ask if they will receive you at this institution—that is if they can receive a voluntary patient." Then I send him there with a note, and in very many cases, after medical treatment, the man is discharged recovered. This is of immense importance, and the more one can get it recognised that these places may be used like homes, the more we shall remove the dread of such institutions, and the people will feel that they are going there for treatment and not for detention. It is the restriction of liberty they regret; it is the restraint the patient feels, and not the surroundings. It is not so much that the patient has people who are more insane about him, but the sensation that he is himself no longer a free agent.

### Boarding Out Patients.

Further on the next line of development of the insane—again following treatment on Scotch lines—is boarding out. In Scotland all over the country they have a patient here and there, or, perhaps, two patients in this or the other small house. In regard to the class of patients, it should be remembered that many people who have been acutely insane recover up to a certain point.

Many hospital cases, both surgical and medical, who have had a severe illness have to leave the hospital only partially recovered, one with a wooden leg, another with a contracted liver; these persons fulfil functions in society and are recognised by society. So on the mental side, an insane person may be left lame in mind, he may, I daresay, no longer be able to fill the precise position he did, being more or less weak-minded, but he is perfectly innocent and harmless; he has been a hewer of wood or a drawer of water in the asylum, he has done creditably a very large amount of mechanical work. I remember a specialist in Bethlem who would do nothing but polish brass rods, another man who still believed himself to be the Holy Ghost, was content with cleaning windows. To a certain extent these people reduce the cost of maintenance, and, therefore, some of the asylum superintendents would not like them to be removed. But it seems to me that the Scotch system is preferable, and one which, instead of increasing asylums to an unlimited extent when such people become chronic, they are boarded out in various villages and village homes and live happier and quieter and less costly lives unless unduly restricted.

There is no doubt that the same sort of thing is developing in England, and here again its manner of developing is rather interesting. In 1890 it was decided that there should be no fresh licenses granted for fresh asylums. The result has been that these hospitals have grown and are receiving a large number of patients who used to be sent to the smaller cheaper private houses. A certain number of these have practically been squeezed out altogether, but there is a revival, and just now hundreds of people all over the country are asking to have patients. Nurses who have married start private asylums and, would you believe it, only about 3,000 doctors have applied to me for patients? Therefore, I presume there is a very large number scattered about. There is a great danger of abuse. In the old days before lunacy legislation was so efficient there were many abuses. Patients were merely, as it were, providers of means for other people to live on. People lived upon lunatics, farming them. That, of course, is the danger that is in front of us; we do not want so much certification of the insane as we want notification. Let there be notification of the insane just as there is notification of fever—you cannot have a typhoid case or a case of small-pox placed anywhere without notification—and so it should be with a person who is insane. If he is a danger to society or to himself let him be put in some specially arranged home, or whatever you like to call it, and let there be a recognition of the fact that these people require some kind of supervision, otherwise I feel sure that abuses will come in. On that line I have found certain so-called colonies very helpful. I am afraid that with our imperial views we are inclined to think that everything is a colony—epileptic colonies, inebriate colonies, feeble-minded colonies, and other useful colonies. One doctor has a farm of 1,000 or 1,200 acres. On that he has ten or a dozen small villas or cottages. In each one of these he has a farm bailiff, a cured inebriate, or some one who has experience himself in mental disorder, and two or three border-line cases, perhaps aphasies, who have given way to drink or who have fallen out of step with society, and yet are not dangerous. These individuals are absorbed into the colony; they are gradually educated up to self-respect. They know nothing about the payment; that is arranged with the head of each house, and the results are extraordinarily good.

The future treatment of insane people is to put them in healthy conditions, to recognise that insanity is not a disease depending on a microbic organism. It may in some cases depend upon that, but in the very large number of cases the insanity is one of relationship of the individual to his surroundings, and if these are modified he may be brought back to usefulness.

### The Sea Voyage and Rest.

Now we come to more particular instances of treatment. We are all inclined to sway backwards and forwards. First it was thought that every lunatic must be shut up. Now the feeling seems to be that every lunatic ought to be made to emigrate. It is said that the path of the Atlantic is being paved by these people. It is a common thing to suggest a sea voyage. It is a dangerous experiment both for the individual and for the attendant. A common occurrence is that after being at sea for a short time the insane person will attempt to drown himself, and the consequence is that the rest of the sea voyage will have to be passed by the individual in his cabin under close supervision. Doctors have in common with others the weakness of recommending that which suits themselves. A yachtsman cannot enter into the feelings of a person who finds sea travelling deadly dull. Travelling often does real harm. If one has an inflamed eye he does not go into a picture gallery to look at pictures in the brightest possible light. And when a man is suffering from painful mental impressions and brain pain, often the best thing to do is to rest. You have to judge what is rest. One finds rest in golf, another by the seashore, and some in pottering about a farm. A distinguished man showed me a road that he had made entirely from beginning to end as a cure for melancholy. It is a most disastrous thing in such cases merely to apply stimulants and movements and attempt to make the patient "buck up." In some people that treatment may be useful. Suppose, for instance, a man makes a fortune by middle age and retires from business. He has devoted himself for 20 years to nothing but money-making. He has not got a healthy vice about him. He cannot play a game or anything else. If you cannot get him back to some business, then travelling will probably settle him down and make a different man of him. Travelling is useful sometimes in the case of the hypochondriac.

When the insanity depends upon some physical disorder like phthisis, the patient ought to be treated for the physical disease. That is what one wants to impress. Treat the individual, not the insanity. It may be the best thing possible for his insanity to send him to a watering-place, such as Aix or Marienbad, treating the man, not the malady. Travelling has its advantages, but it has its grave dangers, and I have seen very many people made worse by travelling, and a great many cases that have ended fatally as the result.

Then comes the pendulum swinging the other way in the hospital treatment for the insane. Give them all rest-cures. Let them rest for three weeks, nine weeks, or three months. If the patient is badly nourished, his digestion failing, if he is generally and physically weak, then the Weir-Mitchell treatment may be of enormous advantage. But if the patient is indolent, and he or she is put to bed simply to soak in indolence, it is the worst thing possible. Brooding leads to hatching, and brooding in bed leads to the hatching of all sorts of delusions. I have seen the most serious results follow on this treatment. I have seen patients put to bed slightly depressed and get profoundly deluded. Under certain conditions, however, it is very beneficial. One recent example of this, a lady suffering from hypochondria, quarrelsome, a pest to the whole household, and nearly two stones and a half below her normal weight, was sent to a nursing home in order to isolate her from her friends. Nurse No. 1 did not get on with her, but the second one did. She was fed, was not seen much by the doctor. The massage was increased, and she regained her two and a half stone and began to think she was getting too fat, and said she was exceedingly happy here. Now comes the time to break down adhesions. She is sent away to the home of a cultured lady who has had experience of such cases, and slowly she gets perfectly well. In such cases the rest-cure did good, but that girl would have gone on having the rest-cure. I have known a patient not leaving the room for 20 years, living in retreat.

### Drug Treatment.

And one must not ignore the treatment by drugs. People nowadays are rather inclined to disparage drugs and drug treatment, but there is no doubt that they are essential in some cases of mental disorder.

They may prevent a breakdown, or they may alleviate in one way or another. I remember the day when patients were kept quiet by antimony and purges. They were made sick or they were purged, and thus kept quiet. We have got past all this, and it is absolutely necessary to remember that purges may be essential and necessary. I have known a person, after a very free action of the bowels, free from delusions. The whole thing seemed to be dependent upon a loaded condition of the bowels. But in treating the insane, do not purge them for purgation sake. In the same way, in relation to sedatives, perhaps there is a useful and a healthy dread of sedatives. I suppose in the case of half the patients I see now the doctors say, "I do not give him anything to make him sleep." That may be right in principle, but rigid principles are inapplicable. You have only to dogmatise on one thing, and you will find that it is not absolutely true. If you find a patient suffering from sleeplessness, it is often your duty to give him sleep in one form or another. Veronal and some new hypnotics are in great favour just now. The bromides are equally good. Chloral is much less used than it was. If veronal does not give sleep, try something else. Before trying any of these it is sometimes quite easy to get sleep by altering the diet, and lessening the amount of tea or coffee. If the patient takes a meal at six or seven and goes to bed at 10.30 with no more food, give him a hot supper with a little stimulant in. It is a very common thing to find old age relieved by stimulant. I always think of a man who told me he was trying my favourite remedy. I asked, "What is that?" He said, "Rum and milk." I suppose this was because I had sometimes ordered it. In those old patients who are emaciated and badly nourished you may try feeding and stimulating together. One thing you have to remember is that if you give them a stimulant and food the last thing at night, it sometimes produces sleep for a couple of hours, after which they wake up and want something else. Rather than a repetition of the stimulant a little milk or something of that sort has a good effect.

### Bath Treatment.

As to baths, they used to be very much used, indeed, in two or three different ways. An old brutal treatment was the surprise bath. The individuals were allowed to sit over a kind of trap-door, and at a signal the trap-door opened and the person went into the bath. The next development was very much the same thing—the shower bath. In my earlier days at Bethlem it was common for the attendant to ask, "Shall I give him a shower?" It was practically a punitive bath. That sort of thing must not be done. In a certain number of patients, especially adolescents, a warm or even hot bath with cold affusions to the head and neck is very often useful; and, again, the most violent maniacal patients I have seen have been benefited by the prolonged bath. You have to fill the bath, and on the bottom of it put two cushions or a pillow filled with hay or straw. The temperature may be varied from a tepid to a warm bath. The patient is put into it, arrangements being previously made to have a strong sheet over the top of it and something to hold the sheet down. During the time the patient is there he will chatter and behave badly, but slowly he will quiet down, after lying for eight or nine hours, as is done at Bethlem. It acts well in some cases of chronic sleeplessness, especially if some mustard tea is put into it, prepared by getting a quarter of a pound of mustard and adding a quart of boiling water to it. Let it stand, and then pour it into the bath. At the end of five or ten minutes the skin gets a little red, and the patient very often sleeps after that when drugs have failed entirely. Turkish baths are often very useful.

### Saline Injections.

One of the most recent treatments is the injections simultaneously of saline fluids, sometimes into the bowel, not infrequently under the skin. In melancholia cases they have been extremely useful. In the treatment of the insane, one thing you have to remember is that you are justified in answering a fool according to his folly; to reason with the unreasonable does little good. Words spoken in front of insane people may be golden or may be leaden. Many years ago I had a lesson I never forgot; an old lady in Bethlem, with wild shaggy hair. I remarked to the attendant that she was like the British lion, and thought no more about it. She got well, and came back to me to tell me that it was not a gentlemanly thing to say that she was like the British lion. About the same time I said to another person, "Can't you behave like a lady." She told me afterwards that it impressed her that I considered it was possible for her to behave differently.

### Hypnotism.

As to hypnotism, this can be useful, but at present we in England do not know very much about it. There are certain cases where one is sure it would do harm—in some of the emotional cases, for example, who believe that people are influencing them already. There are individuals who cannot sleep, and in one of these unfavourable cases I did not think anyone could put her to sleep. She was, however, hypnotised at the very first attempt, and had the first healthy sleep for weeks. In the same way delusions have been shifted by hypnotic suggestions, and will-power undoubtedly strengthened. Inebriates and morphia takers have undoubtedly improved. Be cautious in suggesting which cases are likely to do good and which are not. The eighth volume in Professor Clifford Allbutt's book on the treatment of insanity gives a summary of the experience of Dr. Bramwell, of Goule.

One has to remember that not only have we to treat insanity, but society at present is very much alert as to the future—the prophylaxis. We have not got as far as our American friends, and I do not think we ever shall. It is scarcely to be expected that the passing of laws similar to the one enacted in Minnesota will in any degree do away with the possibility of marriages of those who are mentally defective. Hence we are left with only two methods—ample provision for the poor unfortunates in institutions, or, if they be left out, by castration.

